# An Overview of Sarcopenia: Focusing on Nutritional Treatment Approaches

**DOI:** 10.3390/nu17071237

**Published:** 2025-04-01

**Authors:** Michele Barone, Palmina Baccaro, Alessio Molfino

**Affiliations:** 1 Gastroenterology Unit, Department of Precision and Regenerative Medicine, University of Bari, Policlinic University Hospital, Piazza G. Cesare 11, 70124 Bari, Italy; mina.baccaro@libero.it; 2 Department of Translational and Precision Medicine, Sapienza University of Rome, 00185 Rome, Italy; alessio.molfino@uniroma1.it

**Keywords:** muscle mass, muscle strength, nutrients, omega-3 fatty acids, β-hydroxy-β-methylbutyrate, vitamin D, branched-chain amino acids, whey proteins

## Abstract

Sarcopenia is a syndrome characterized by the progressive and generalized loss of skeletal muscle mass and strength. This condition is associated with physical disability, decreased quality of life, and increased mortality. Therefore, reducing the prevalence of sarcopenia could significantly lower healthcare costs. Sarcopenia can be classified into primary and secondary sarcopenia. The former is related to aging and begins after the fourth decade of life; after that, there is a muscle loss of around 8% per decade until age 70 years, which subsequently increases to 15% per decade. On the other hand, secondary sarcopenia can affect all individuals and may result from various factors including physical inactivity, malnutrition, endocrine disorders, neurodegenerative diseases, inflammation, and cachexia. Understanding the multiple mechanisms involved in the onset and progression of sarcopenia allows for us to develop strategies that can prevent, treat, or at least mitigate muscle loss caused by increased protein breakdown. One potential treatment of sarcopenia is based on nutritional interventions, including adequate caloric and protein intake and specific nutrients that support muscle health. Such nutrients include natural food rich in whey protein and omega-3 fatty acids as well as nutritional supplements like branched-chain amino acids, β-hydroxy-β-methylbutyrate, and vitamin D along with food for special medical purposes. It is important to emphasize that physical exercises, especially resistance training, not only promote muscle protein synthesis on their own but also work synergistically with nutritional strategies to enhance their effectiveness.

## 1. Introduction

This narrative review aims to highlight key aspects of sarcopenia that are useful to explain how a nutritional approach can contribute to treating this clinical condition. We will begin by explaining the differences between primary and secondary sarcopenia along with the physiopathological mechanisms involved in their development. In the second part of the review, we will discuss possible metabolic pathways mediated by specific foods and nutritional strategies to improve or prevent sarcopenia. By enhancing the understanding of the mechanisms supporting the beneficial effects of nutrients, we hope to promote the dissemination and development of new targeted clinical strategies that can help mitigate the debilitating and health-threatening effects of sarcopenia.

The search strategy of the scientific articles found in the literature is described in Supplementary Tables S1 and S2.

## 2. Definition

The term “sarcopenia”, derived from the Greek words “sarx” (meaning muscle) and “tenia” (meaning loss), was first introduced by Irwin Rosenberg. He defined sarcopenia as a progressive loss of skeletal muscle mass associated with aging [1,2]. In 2010, the European Working Group on Sarcopenia in Older People (EWGSOP) revised this definition to describe sarcopenia as a syndrome characterized by the progressive and generalized loss of skeletal muscle mass and strength. Sarcopenia has significant negative effects on health, leading to physical disability, a diminished quality of life, and an increased risk of mortality [3]. The Asian Working Group on Sarcopenia adopted a similar definition but established different diagnostic cutoffs tailored to the Asian population [4]. In 2018, the criteria for identifying sarcopenia were updated again by the same working group. In 2018, the same working group (referred to as the EWGSOP2) updated the criteria for identifying sarcopenia. This update emphasized muscle strength reduction as the primary diagnostic parameter, as muscle strength is considered a more reliable indicator of muscle function [5]. Sarcopenia has been officially recognized as a muscle disease in the International Classification of Diseases (ICD-10). The code M62.84 is designated for age-related sarcopenia, while code M62.8 is used for sarcopenia linked to other muscle conditions. However, there is not a widely accepted definition of sarcopenia, and three of them represent updates to the original definitions [6].

The EWGSOP2 guidelines outline the criteria for identifying the progression of muscular damage (see Table 1). Sarcopenia is considered probable when there is evidence of low muscle strength; a definitive diagnosis occurs when low muscle strength is associated with low muscle mass. Additionally, a severe degree of sarcopenia is characterized by a combination of low muscle strength, reduced muscle quantity or quality, and clinical evidence of decreased physical performance.

The rationale for using two criteria for diagnosing sarcopenia stems from evidence indicating that muscle strength is not solely dependent on muscle mass. Although there is a correlation between strength and mass, it is not linear [7,8]. This means that increases in muscle mass do not necessarily result in proportional increases in strength. Therefore, defining sarcopenia solely in terms of muscle mass is too restrictive and may have limited clinical value [3].

## 3. Classification

Sarcopenia mainly occurs in older individuals, but it can also develop in young adults. In clinical practice, two types of sarcopenia are typically recognized: primary and secondary sarcopenia (see Table 2). Primary sarcopenia is diagnosed when aging is the only apparent cause, whereas secondary sarcopenia occurs when there are identifiable contributing factors. These factors can include reduced physical activity, malnutrition, endocrine disorders, decreased production of or sensitivity to anabolic hormones, neurodegenerative diseases, dysregulation of cytokine secretion, the presence of an inflammatory state, and cachexia [3].

Secondary sarcopenia can be further divided into acute and chronic. Acute sarcopenia lasts for less than 6 months and is typically associated with a sudden illness or injury. In contrast, chronic sarcopenia persists for 6 months or longer and is often linked to ongoing, progressive pathological conditions [5].

## 4. Epidemiology

According to the definition reported in the EWGSOP2 guidelines, sarcopenia affects approximately 10–20% of individuals over the age of 60 years and about 30% of those over 80 years. In Europe, the number of people affected by sarcopenia is expected to rise significantly in the future due to the increase in the average life expectancy of the population. This represents a socioeconomic problem worsened by the coexisting increased trend of obesity [9]. Moreover, an opposite trend is observed in physical activity, which diminishes its beneficial effects on muscle health. This may lead to a positive energy balance and weight gain, represented mainly by fat. The loss of muscle mass further reduces the amount of insulin-responsive tissue, contributing to insulin resistance. This condition can promote the development of metabolic syndrome and obesity [10]. The increased fat mass is responsible for the production of inflammatory markers, such as tumor necrosis factor-alpha (TNF-α), interleukin-6 (IL-6), and other adipokines, which not only exacerbate insulin resistance but also have a direct catabolic effect on muscles. All these changes lead to the development of a condition defined as sarcopenic obesity, which consists of a reduction in lean body mass associated with excess adiposity [11], more commonly observed in older individuals [12]. Obesity worsens the effect of sarcopenia, leading to increased fat infiltration in muscles, reduced physical function, and higher mortality risk [13,14]. Notably, a concerning trend regarding obesity has also been observed in patients undergoing liver transplantation [15].

Sarcopenia can occur before the age of 60 years, particularly in specific clinical settings such as cancer [16], kidney dysfunction [17], liver disease [18], metabolic disorders [19], and rheumatological diseases [20]. In these contexts, it represents a prognostic indicator for potential complications and survival [16,19].

It is estimated that a 10.5% reduction in the prevalence of sarcopenia could decrease annual healthcare costs by $ 1.1 billion in the United States [21]. However, a significant limitation in studying the prevalence of sarcopenia depends on the varying definitions used for its diagnosis, the age of the study group, the clinical setting, and the method used for its assessment [22,23,24].

## 5. Physiopathological Aspects

There are numerous mechanisms involved in the onset and progression of sarcopenia. These mechanisms encompass protein synthesis, proteolysis, neuromuscular integrity, and muscle fat content, which are all integral to the aging process.

Muscle synthesis is intrinsically linked to muscle atrophy via the PI3k–Akt pathway, which is central to both processes [25]. It is activated by the binding of insulin-like growth factor (IGF-1) to its receptor, which triggers the PI3k–Akt–mTOR signaling pathway, promoting protein synthesis [26]. The binding of IGF-1 to its receptor activates its intrinsic tyrosine kinase in muscle cells, leading to autophosphorylation and subsequent phosphorylation of the insulin receptor substrate. This substrate then acts as a docking site to recruit and activate phosphoinositide-3-kinase (PI3K), which phosphorylates membrane phospholipids, generating phosphoinositide-3,4,5-trisphosphate (PIP3). PIP3, in turn, serves as a docking site for two kinases, phosphoinositide-dependent kinase 1 (PDK1) and Akt; the subsequent phosphorylation of Akt by PDK1 activates it. The activation of Akt is a critical step for muscle growth, as it inhibits protein degradation by phosphorylating and thus suppressing the Forkhead Box (FoxO) family transcription factors and stimulates protein synthesis via mammalian target of rapamycin (mTOR) [27]. mTOR subsequently stimulates muscle protein synthesis by phosphorylating various targets, including the translation initiation factors S6K1 and 4E-BP1, which promote the translation of mRNA involved in muscle protein synthesis. In a state of balance, protein synthesis and degradation are balanced to maintain muscle mass.

**Figure 1:**
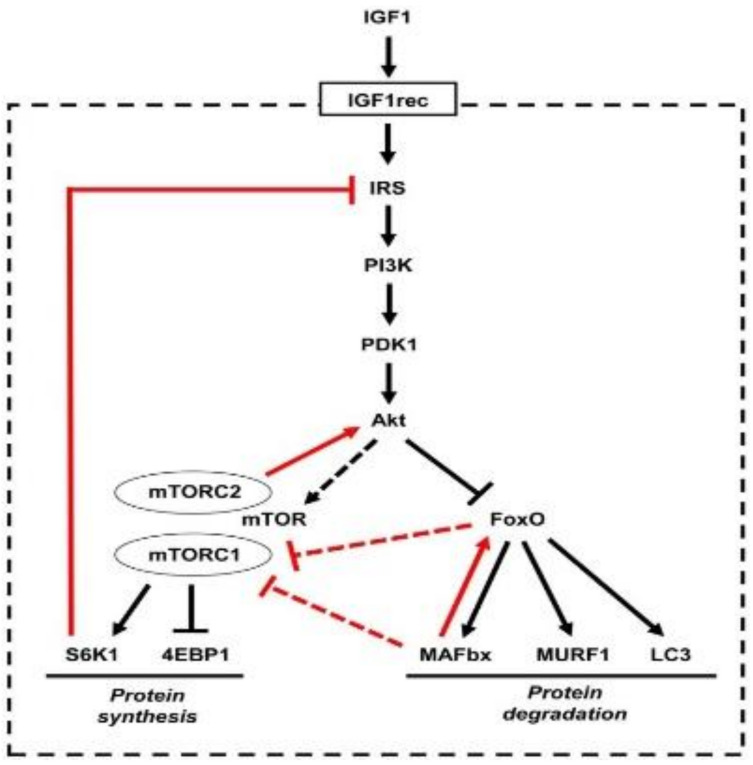
The insulin-like growth factor 1 (IGF1)–Akt pathway that controls muscle growth via mammalian target of rapamycin (mTOR) and muscle degradation via FoxO. Reproduced with permission from Schiaffino et al. [25].

FoxO regulates the expression of the ubiquitin ligases atrogin-1 (also called muscle atrophy F-box or MAFbx) and muscle ring finger 1 (MuRF1), which control the ubiquitination of myosin and other muscle proteins, targeting them for degradation by the proteasome [26]. The phosphorylation of FoxO by Akt prevents its action in the nucleus, thereby inhibiting muscle degradation (Figure 1) [28,29,30].

However, external stimuli, such as physical exercise and nutrients, can shift the balance towards anabolism, while stress, inactivity, and aging can promote catabolism, leading to muscle loss. During the natural aging process, protein synthesis tends to decrease due to a reduction in the activation of the IGF1–Akt/mTOR pathway, while the activity of catabolic processes mediated by FoxO increases. This imbalance leads to a gradual loss of muscle mass and strength, known as primary sarcopenia.

Most studies investigating sarcopenia in humans suggest that the loss of muscle mass is attributable to a phenomenon known as “anabolic resistance”, which refers to the body’s diminished ability to efficiently use dietary proteins to promote muscle protein synthesis, a process essential for maintaining and building muscle mass [31,32,33]. Catabolic pathways can be particularly activated during extreme inactivity, such as bed rest, and chronic inflammatory conditions. A recent study involving five days of bed rest in both young and elderly healthy subjects found that lean mass and leg strength were reduced only in the elderly participants. After bed rest, both groups had attenuated mTORC1 signaling and increased MURF1 expression in skeletal muscle, but only the older group showed a reduction in amino acid-induced protein synthesis rates and an increase in atrophy factors [34]. Similarly, a study examining the effects of resistance training in participants with chronic obstructive pulmonary disease demonstrated higher levels of MAFbx and MURF1 and increased phosphorylation of S6K1 kinases, determining its deactivation [35].

Recent studies have highlighted that sarcopenia is not merely a consequence of aging but also serves as a key mediator of low-grade chronic inflammation (inflammaging). This determines a vicious cycle that accelerates the physical and functional decline in older adults [36,37].

Muscles play a critical role in various metabolic processes that can improve health or increase the risk of diseases. They are the primary site of insulin-stimulated glucose disposal and constitute the largest component of basal metabolism. Muscles affect either directly or indirectly bone density, produce myokines with pleiotropic effects on muscle and other tissues including the brain, and act as a reserve of essential amino acids for protein synthesis during periods of reduced food intake and stress [38]. Therefore, it is not surprising that the decline in skeletal muscle health is a powerful risk factor and the primary consequence of chronic diseases, disability, and mortality. However, skeletal muscles are one of the most adaptable tissues in the body; they can quickly change their in-protein synthesis and degradation rates in response to physical activity, inactivity, inflammation, as well as nutritional and hormonal factors [38].

Skeletal muscles also act as endocrine organs by secreting hormone-like factors known as myokines. Released by muscles during contraction, myokines are proteins that play a crucial role in regulating inflammation and metabolism, particularly concerning aging and sarcopenia. Like adipokines, cytokines, and other factors released by adipose tissue, myokines significantly contribute to these processes [39]. As people age, the production of myokines changes significantly, affecting both muscle health and overall metabolism. Moreover, reduced physical activity, commonly associated with aging, exacerbates this effect. Consequently, the production of beneficial myokines, such as irisin and IL-15, tends to decrease, while the levels of catabolic myokines, such as myostatin, increase. This imbalance contributes to the development of sarcopenia. Additionally, the chronic low-grade inflammation that characterizes aging, also known as “inflammaging”, may further reduce myokine function, creating a vicious cycle that accelerates both muscle and metabolic deterioration [40].

*Myostatin*, also known as “growth and differentiation factor 8” (GDF-8), is a protein belonging to the TGF-β family and acts as a negative regulator of muscle growth. Discovered in 1997, it is primarily produced in skeletal muscles but is also found in cardiac muscles and adipose tissue [41,42]. Studies in animal models have shown that inhibition of myostatin leads to a significant increase in muscle mass, which has driven the development of therapies based on myostatin antagonists to treat muscle atrophy conditions. However, attempts to use such therapies in humans have faced difficulties, with many candidate drugs failing to achieve the desired clinical results [40]. Although the expression of myostatin increases with age, Wilhelmsen et al. have suggested that skeletal muscle myostatin mRNA expression increases only in older adults with excess adiposity rather than due to insulin resistance or aging itself [43].

*Irisin* is a myokine produced and released by skeletal muscles during physical exercise [44] and promotes the transformation of white adipocytes into beige adipocytes, which are more metabolically active and contribute to thermogenesis and regulation of energy metabolism [45,46]. Irisin also promotes mitochondrial biogenesis and oxygen consumption in muscles and is inversely correlated with myostatin, suggesting its role in promoting muscle growth [40]. Additionally, lower levels of irisin have been found in women with post-menopausal sarcopenia [47], suggesting its potential pro-myogenic role in pathological conditions. This myokine also induces muscle hypertrophy and attenuates muscle atrophy, suggesting a possible protective effect against sarcopenia and muscle aging through the activation of satellite cells and protein synthesis [40].

Based on these premises, an interesting study investigated the role of irisin as a possible protective factor in Charcot-Marie-Tooth disease (CMT), which is characterized by a progressive neuropathy responsible for gradual muscle atrophy and decreased muscle strength, ultimately leading to motor disabilities [48]. Patients with CMT had lower skeletal muscle mass and significant irisin level reduction than age- and sex-matched healthy subjects; moreover, patients with a larger reduction in muscle strength had significantly lower irisin levels than those with less compromised muscle strength.

*Interleukin-6 (IL-6)* is a cytokine with pleiotropic functions, depending on the biological context. IL-6 has a pro-inflammatory role and is involved in immune response and inflammation. On the other hand, this cytokine also has anti-inflammatory properties when released by muscles during physical exercise, regulating the production of other pro-inflammatory cytokines such as TNF-α [39]. Paradoxically, IL-6 is associated with either growth or atrophy in skeletal muscle mass regulation [49]. Interestingly, there is an inverse relationship between regular physical activity or endurance training and plasma IL 6 levels, whereas the opposite is observed with physical inactivity. Notably, the expression of the IL 6 receptor (IL-6Rα) is strikingly upregulated in response to endurance training [39]. When released by muscles during exercise, IL-6 has a modulatory effect on energy metabolism, promoting fat oxidation and improving insulin sensitivity through various signaling pathways [40]. In chronic conditions, such as age-related muscle atrophy or diseases like Duchenne muscular dystrophy, IL-6 can have a negative effect, contributing to muscle mass loss through inflammatory mechanisms [50,51]. Therefore, inhibiting IL-6 activity has been explored as a potential therapeutic strategy to mitigate muscle decline in these conditions [40].

*Interleukin-15* (IL-15), a cytokine structurally similar to interleukin-2 (IL-2), was discovered in 1994 as a growth factor for T cells. Subsequent studies revealed that IL-15 accumulates in muscles following exercise, indicating its role as a myokine [52,53,54,55]. Several in vitro studies have shown that IL-15 stimulates the differentiation of myoblasts and increases muscle mass in murine skeletal muscle cells [56,57]. Additionally, IL-15 activates the Jak3/STAT3 signaling pathway to mediate glucose uptake in skeletal muscle cells [58].

In rats with cancer cachexia, IL-15 treatment reduces muscle breakdown through the inhibition of the ATP-dependent ubiquitin–proteasomal pathway [59]. In pancreatic cancer, the IL-15 production stimulated by exercise promotes tumoral infiltration of CD8+T cells, CD8+T cell survival, and a cytotoxic/effector phenotype [60,61].

*Decorin* is a small leucine-rich proteoglycan that is secreted in skeletal muscles during muscle contraction, playing an important role in muscle growth [30]. Interestingly, decorin directly binds to myostatin and inactivates it in a zinc-dependent manner, inhibiting its anti-myogenic effects [62]. In a study conducted by Marshall et al. [63], overexpression of decorin increased the expression of myogenic differentiation 1 (Myod1) and follistatin. Both Myod1 and follistatin are key factors involved in the regulation of muscle growth and regeneration. Myod1 is a transcription factor that induces the differentiation of muscle stem cells (myoblasts) into mature myocytes, promoting the expression of muscle-specific genes necessary for the formation and maintenance of skeletal muscle tissue, thus being essential for muscle development and repair after injury [64,65,66]. More recently, it has been proved that decorin, activin A, and follistatin are increased by moderate-to-high-intensity interval resistance training in obese patients with cardiovascular risk [67]. On the other hand, follistatin not only inhibits the activity of myostatin, thereby promoting muscle growth, but it can also block other TGF-β family proteins that limit muscle tissue growth, promoting both the proliferation and differentiation of muscle cells [68,69,70]. Based on these considerations, we can assert that decorin may act as a myogenic factor and could represent a potential therapeutic target for the treatment of muscle atrophy.

## 6. Primary Sarcopenia: The Physiological Process of Aging

The decrease in skeletal muscle mass with aging has a complex etiology, which involves molecular, neurological, cellular, and metabolic factors associated with a progressive loss of motor neurons (up to 50% of motor units) by the eighth decade of life [71,72]. It is important to note that skeletal muscle consists of two types of fibers: type I and type II fibers. The latter have a higher glycolytic potential, lower oxidative capacity, and a faster response compared to type I slow fibers. Type I fibers are known as fatigue-resistant fibers due to their characteristics, including a higher density of mitochondria, capillaries, and myoglobin content. Most muscles contain both fiber types, except for postural muscles, which are composed of type I fibers only. During slow and low-intensity activity, most of the force generated by muscle contraction comes from type I fibers, while in high-intensity exercise, both type I and II fibers are equally involved. Larsson et al. [73] described a selective atrophy of type II fibers with a relative preservation of type I fibers as aging progresses. The multiple pathways leading to reduced rates of muscle protein synthesis with age include increased insulin resistance (secondary to increased body fat and inactivity), inflammation, reduced testosterone and estrogens, and decreased growth hormone production. Moreover, the functional impairment of mitochondrial function determines a reduction in energy production and elevated levels of intracellular oxidative stress, contributing to DNA damage, which accelerates muscle loss by apoptosis and produces skeletal muscle atrophy. Finally, epigenetic modifications, such as DNA methylation, have been recognized as contributing factors to primary sarcopenia [38,74].

**Figure 2:**
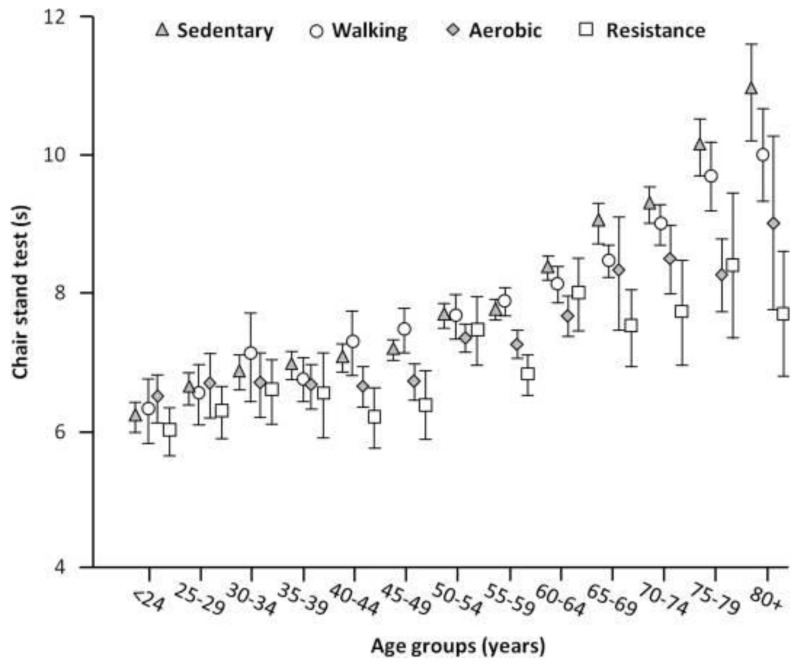
Time needed to complete the “Chair stand test” based on different types of physical activity and age groups. Reproduced with permission from Landi et al. [78].

An important cause of primary sarcopenia is inactivity [75]. It is worth noting that ten days of bed rest in healthy older adults (67 ± 5 years) resulted in a 30% reduction in the muscle protein synthesis rate, with a loss of nearly 1 kg of lean leg mass and a 15.6% loss in leg strength [76]. This loss of lean leg mass is nearly three times greater than that observed in healthy young men and women after 28 days of bed rest [34,77]. These dramatic losses of muscle mass in such short periods, typical of hospitalizations, often result in the catastrophic loss of strength, functionality, and independence. A study by Landi et al. [78] examined the impact of habitual physical activity and different types of exercise on muscle performance in 6242 adults aged 18 to 98 years (mean age 54.4 years; 57% women). The results, measured by the “chair stand test” to evaluate muscle performance, showed that, between 18 and 44 years of age, muscular performance remained stable regardless of lifestyle, and then, it progressively declined with age. However, physically active participants completed the test, on average, 0.5 s faster than sedentary individuals (*p* < 0.001). Furthermore, the intensity of physical activity played a decisive role in the decline of performance, which was similar between sedentary participants and those who only walked for leisure, while it was significantly attenuated in participants engaged in aerobic or resistance training. In particular, octogenarians who used a combination of aerobic and resistance training achieved performance comparable to individuals aged 50–54 years (Figure 2). In general, the progressive loss of muscle mass that begins around the age of 40 years is about 8% per decade until the age of 70 years, after which the loss increases to 15% per decade [79].

Aging is associated with changes in hormone production and sensitivity, particularly regarding growth hormone (GH), insulin-like growth factor-1 (IGF-1), corticosteroids, androgens, estrogens, and insulin [3]. The decrease in sex hormones with age helps explain the gender differences in the development of age-related sarcopenia. However, the mechanisms behind these effects vary. It is widely accepted that the decline of testosterone is one of the most important factors directly responsible for the loss of muscle mass and strength in men [80]. In menopausal women, the reduction in estrogens leads to an accumulation of visceral fat. This occurs, in part, due to a relatively higher increase in leptin and a decrease in adiponectin compared to men. These changes can lead to increased appetite and a decrease in anti-inflammatory and insulin-sensitizing activity, ultimately contributing to the development of sarcopenia [81,82]. Moreover, the reduced modulatory effects of sex hormones on mitochondrial function also play a role in the onset and progression of sarcopenia [83].

It is now widely recognized that low levels of vitamin D in the blood are associated with a decrease in muscle strength. In fact, it has recently been shown that vitamin D deficiency or insufficiency is positively correlated with the risk of several diseases, including sarcopenia, cardiovascular diseases, obesity, and cancer; specifically, biological, clinical, and epidemiological evidence has been examined to support the association between vitamin D and an increased risk of sarcopenia in older adults [84].

## 7. Secondary Sarcopenia

Secondary sarcopenia is linked to three main pathogenetic mechanisms: inflammatory activity, impaired physical activity, and nutritional deficiencies [3]. While there is a widely accepted definition of primary sarcopenia, for secondary sarcopenia, there is still no consensus both in research and clinical practice [5,85]. Sarcopenia can be determined by acute pathological conditions (surgical interventions, wounds, polytrauma, sepsis, burns, etc.) or chronic diseases in active phases, which are conditions associated with an increased energy expenditure and hypercatabolism. These conditions produce metabolic dysregulations, with an increase in protein catabolism that significantly affects the body’s largest protein reserve, skeletal musculature [86]. In these cases, amino acids are destined for various uses, including acute-phase protein synthesis, proteins involved in the immune response or wound healing, and energetic production. It has also been observed that hepatic gluconeogenesis, stimulated during stress, requires additional amino acids, largely sourced from muscle tissue [87]. This muscle degradation is difficult to reverse, even with aggressive nutritional support. In cancer patients, additional mechanisms that are related to tumor growth are involved in muscle mass loss.

The reduced physical activity due to immobilization or disability related to illness contributes to the development of sarcopenia [88]. Finally, sarcopenia can develop due to inadequate energy and/or protein intake, resulting from conditions such as anorexia, malabsorption, limited access to healthy foods, or feeding difficulties [5]. Finally, in patients with neurodegenerative diseases, the loss of muscle mass and function is an integral part of the disease [89].

It should also be noted that the mechanisms leading to sarcopenia are multiple and interconnected, making it difficult to distinguish the primary causes for each individual patient.

When muscle loss is combined with inflammation, it is defined as “cachexia”, a condition present in several pathological conditions (cancer, heart failure, COPD, chronic kidney disease, inflammatory bowel diseases, rheumatoid arthritis, HIV, liver cirrhosis, etc.) [90]. Therefore, although “sarcopenia” and “cachexia” are characterized by loss of muscle mass and strength, they differ in origin and pathophysiological mechanisms. “Cachexia” is characterized by a complex metabolic syndrome that involves increased muscle catabolism (negative protein–energy balance), independent of nutritional intake, and is linked to a systemic inflammatory state that promotes muscle protein breakdown. Unlike sarcopenia, cachexia is less reversible, even with nutritional support, and represents a significant negative prognostic factor [91]. The trajectory of a patient with chronic disease is closely related to changes in body composition (loss of muscle mass and alterations in adipose tissue) and metabolic dysfunctions that can impact the outcome of the disease itself. These changes not only reduce the physical strength and autonomy of the patient but also compromise immune function, increasing the risk of infections and worsening the clinical course.

Among the diseases responsible for secondary sarcopenia, one of the most important is the alteration of the mechanisms responsible for nutrient digestion and absorption, as in chronic inflammatory gastroenterological diseases [92,93]. These conditions are characterized by active inflammation that increases intestinal membrane permeability; cause both local and systemic inflammatory effects [94,95]; and impact quality of life, prognosis, and treatment with surgical, biological, and immunomodulatory interventions [92].

It is important to underline that “disease”, “inflammation”, and “inactivity” are variables that are intimately connected, as the pathway to sarcopenia often begins with a chronic disease that triggers systemic inflammation and induces physical inactivity. Disease triggers an inflammatory response that leads to the degradation of muscle proteins, reduction in muscle mass, and alteration of metabolism. Additionally, some conditions, such as rheumatological diseases, can cause not only systemic inflammation but also “physical inactivity” [96]. This reduction in physical activity further contributes to a decline in muscle strength and mass, promoting the onset of sarcopenia, and physical disability, which is one of the main predictors of sarcopenia in rheumatologic diseases [20].

### Cancer-Related Sarcopenia

**Figure 3:**
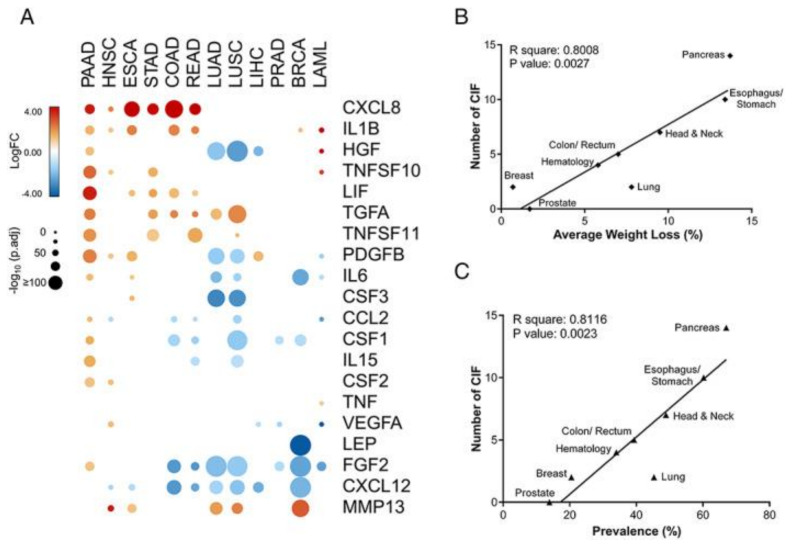
Tumor-specific expression profiles of cachexia-inducing factors (CIFs) and their correlations with the prevalence of cachexia and weight loss. (**A**) Schematic representation of the expression pattern of 25 CIFs in different tumor types of The Cancer Genome Atlas (TCGA); (**B**,**C**) Pearson’s correlations coefficient (r) with corresponding *p* values for the covariation between the number of differentially expressed CIFs (y-axis) from TCGA data sets (tumor tissues vs. matched normal TCGA and GTEx data) and the percentage of weight loss (x-axis; (**B**)) or the percentage of cachexia prevalence (x-axis; (**C**)) for specific tumour types from relevant literature data. Reproduced with permission from Freire et al. [98].

Sarcopenia in cancer represents a unique condition of secondary sarcopenia since it involves specific signals derived from tumor cells that deeply affect cellular metabolism [97]. These signals exacerbate muscle breakdown compared to muscle growth processes, leading to noticeable weight loss, with most patients experiencing significant sarcopenia. In this context, a crucial role is played by cachexia-inducing factors (CIFs), a group of cytokines, growth factors, and secreted proteins that contribute to the dysregulation of muscle metabolism [98]. Cancers of the pancreas, stomach, esophagus, and head and neck are among those with the highest number of CIFs expressed, associated with a higher prevalence of cachexia and more significant weight loss (Figure 3).

It is important to note that cachexia is not present in all types of cancer, while muscle mass loss can occur more frequently across various cancer types, even in the presence of maintained or increased body weight [99,100]. The “pro-inflammatory cytokine storm” that occurs during neoplastic events triggers an energy-costly, acute-phase response with a high demand for essential amino acids. Additionally, tumors inducing cachexia produce lipid-mobilizing factors (LMF), promoting the degradation of adipose tissue and oxidation of fatty acids [101]. Recent evidence suggests that white adipose tissue undergoes a “browning” process, with increased expression of uncoupling protein 1 (UCP1), which diverts energy from ATP synthesis toward thermogenesis, increasing lipid mobilization and energy expenditure. Moreover, the infiltration of adipose tissue into skeletal muscle contributes to reduced muscle density, exacerbating sarcopenia [102].

In gastrointestinal cancer patients, muscle wasting is associated with overactivation of intracellular protein degradation systems, particularly the ubiquitin–proteasome and autophagy–lysosomal pathways, which accelerate muscle protein breakdown. Recent research highlights the involvement of small non-coding RNAs (sncRNAs), particularly muscle-specific microRNAs (myomiRs), which are dysregulated in the skeletal muscle of cancer patients and may contribute to muscle atrophy. Notably, increased circulating levels of myomiRs, such as miR-133a-3p and miR-206, have been observed in male cancer patients with preserved muscularity, indicating a possible compensatory protective response. Additionally, the altered expression of P-element induced wimpy testis (PIWI)-interacting RNAs (piRNAs), particularly the downregulation of piR-12790 and piR-2106, points to a broader epigenetic dysregulation that could impair muscle maintenance in cancer patients. These findings suggest that circulating and muscle-specific sncRNAs may serve not only as biomarkers for cancer-related muscle loss but also as potential therapeutic targets to mitigate cancer-associated sarcopenia [103].

## 8. The Nutritional Treatment of Sarcopenia

The treatment of sarcopenia must necessarily include an appropriate nutritional approach that considers both specific components of the diet (dietary patterns) and nutrient intakes (total calories) [104]. A well-balanced diet, rich in high-quality proteins, vitamins, minerals, and healthy fats, is fundamental in managing sarcopenia. The addition of specific supplements, such as creatine, omega-3 fatty acids, leucine, and beta-hydroxy-beta-methylbutyrate (HMB), can help in reducing the risk and progression of sarcopenia [105]. Moreover, increasing the intake of antioxidants may help reduce inflammation, which is a key factor in the development of sarcopenia [106].

The dietary prescription to counteract sarcopenia should also provide sufficient calories and ensure the correct intake of nutrients based on individual characteristics (age, sex, comorbidities, concomitant therapies, total energy requirements). However, although there is increasing scientific interest in gender medicine [107], nutritional guidelines still do not provide distinct practical recommendations based on gender [108]. The timing of nutrient administration also plays an important role in optimizing the effect of the diet on the treatment of sarcopenia. The endogenous circadian clock coordinates the diurnal variation in most metabolic processes, and an early timed breakfast plays a pivotal role in controlling the expression of peripheral clock genes responsible for enhancing enzymes and hormones involved in glucose metabolism regulation and muscle synthesis [109]. Therefore, an alteration in clock-gene expression is associated with a risk of obesity and sarcopenia, with significant mass loss. In line with the importance of timing in muscle protein metabolism, performing heavy-resistance exercise in the late afternoon affects the circadian rhythm of anabolic/catabolic hormones, such as the testosterone/cortisol ratio, and skeletal muscle protein synthesis [110].

Recent guidelines on protein needs for patients with sarcopenia suggest a protein intake of 1.2 g per kilogram of body weight per day, except in patients with severe renal impairment [111]. However, during periods of acute catabolism (e.g., prolonged bed rest due to illness or inability to engage in physical activity), higher protein intake is crucial to protect skeletal muscle [112].

Various indices have been developed to assess diet quality in older adults, based on the consumption of “healthier” food groups (e.g., whole grains, fruits, vegetables), nutrients particularly relevant for older individuals (e.g., proteins, vitamin D, calcium, vitamin B12, folate, fluids), and the intake of macronutrients and fatty acid ratios [113,114].

Diet quality and dietary patterns are increasingly considered in nutritional studies on sarcopenia. In fact, it has been observed that “high-quality diets” are associated with better physical functionality and a reduced risk of sarcopenia [104,115,116,117]. It is also important to note that “high-quality diets”, often referring to the “Mediterranean diet” and the “Nordic diet”, are frequently associated with the adoption of health-promoting lifestyles [118]. Recent studies indicate that the adherence to the Mediterranean diet can improve muscle strength and function, reducing the risk of developing sarcopenia [119,120]. It is believed that the muscle-protective properties of the Mediterranean diet are related to its balanced content of vitamins E and C, carotenoids, and phytochemicals with antioxidant properties [121]. Additionally, essential fatty acids, particularly omega-3 fatty acids, are involved in muscle metabolism. These fatty acids, including linolenic acid and its metabolic products such as eicosapentaenoic acid (EPA) and docosahexaenoic acid (DHA), found in fish oil, promote muscle anabolism. A high omega-6/omega-3 ratio can lead to higher levels of IL-6, interfering with IGF-1-mediated processes and blocking the phosphorylation of the protein p70s6k, which is necessary for activating protein synthesis [122].

Despite the initial promising results, not all studies confirm the effectiveness of omega-3s in the treatment of sarcopenia. This may be due to several factors such as dosage, intervention duration, initial nutritional status, and individual characteristics of the participants [123]. Douglas Paddon-Jones et al. [124] highlighted that an intake of approximately 25–30 g of high-quality protein per meal represents the optimal level to stimulate muscle protein synthesis, both in young and elderly individuals. However, in older subjects, the muscle anabolic response is reduced when proteins are consumed in combination with carbohydrates or when the protein intake is less than 20 g per meal. Fortunately, it has also been demonstrated that moderate physical activity can sensitize muscles to subsequent nutritional stimuli, restoring insulin’s ability to stimulate muscle protein synthesis [125].

These data emphasize the importance of appropriate physical exercise in enhancing the anabolic response to dietary protein on muscle mass and function associated with aging. To obtain a more detailed picture and identify specific nutritional deficiencies, the data collected through malnutrition screening tools can be supplemented with targeted laboratory tests. Specifically, biochemical markers, such as hemoglobin, ferritin, vitamin B12, prealbumin, creatine phosphokinase (CPK), and vitamin D, can complete the nutritional assessment [126,127,128].

Although some nutrients and dietary patterns seem to have a potential protective effect against sarcopenia in cancer patients, an inadequate protein and energy intake can compromise any dietary strategy; therefore, the addition of oral nutritional supplements (ONS) should be considered as a complementary approach to counteract sarcopenia [129]. However, although such supplementation can play a role in meeting nutritional needs, further research is needed to clarify and confirm its beneficial effects [130].

Another important topic that deserves attention, as it relates to both nutrition and physical activity, is the gut microbiome [131,132,133] A recent meta-analysis examining the effects of probiotics on sarcopenia analyzed 22 RCTs involving 1028 patients from different countries, including fourteen from Southeast Asia, one from Iran, five from Northeastern Europe, and two from the USA [134]. The authors concluded that, although the efficacy of probiotics remains controversial, they can significantly improve muscle strength. However, it is worth noting that only three of the studies received a high-quality evaluation, and 50% of all the participants were overweight or had obesity or metabolic syndrome.

Finally, it is important to specify that any nutritional intervention always requires continuous monitoring to verify the results and adjust the treatment as necessary. This implies clinical, anthropometric, and, if necessary, instrumental evaluations to assess the possible changes in muscle mass and strength.

### 8.1. Specific Dietary Components and Protein Synthesis

#### 8.1.1. Branched-Chain Amino Acids and Whey Proteins

The branched-chain amino acids (BCAAs) leucine, isoleucine, and valine increase skeletal muscle protein synthesis, and their supplementation is used to improve athletic performance and attenuate muscle loss [135]. In particular, leucine supplementation has become increasingly popular due to the discovery of its anabolic effects on cellular signaling and muscle protein synthesis mediated by the mTORC1 complex activation, i.e., the target regulating protein synthesis, cell growth, and metabolism in response to external stimuli, such as nutrients (e.g., amino acids) and hormones [136]. The anabolic stimulus represented by BCAAs can shift the net protein balance from catabolism to anabolism, attenuating muscle loss due to illness, bed rest, or aging [137,138]. In this regard, it should be underlined that whey proteins are the richest source of BCAAs and contain the highest amount of leucine [139]. Whey proteins are rapidly digested and absorbed, producing a high level of aminoacidemia that further increases anabolic processes [140,141,142]. Resistance exercises appear to maximize the effect of BCAA supplementation. A study conducted by Wolfe [143] reports that an amino acid blood level increase stimulates muscle protein synthesis, and the effectiveness of this stimulation varies based on the dosage and mixture of amino acids and the age as well as hormonal profile of the subject. However, despite the increase in muscle protein synthesis, in the fasting state following exercise, there is also an increase in protein degradation. This can lead to a net negative balance between synthesis and degradation, emphasizing that, without a sufficient nutrient intake, the protein balance may remain negative [143]. Finally, it has been shown that there is a synergistic effect between exercise and amino acids, as demonstrated by the net anabolic response obtained with amino acids alone or exercise alone [143].

#### 8.1.2. β-Hydroxy-β-Methylbutyrate

**Figure 4:**
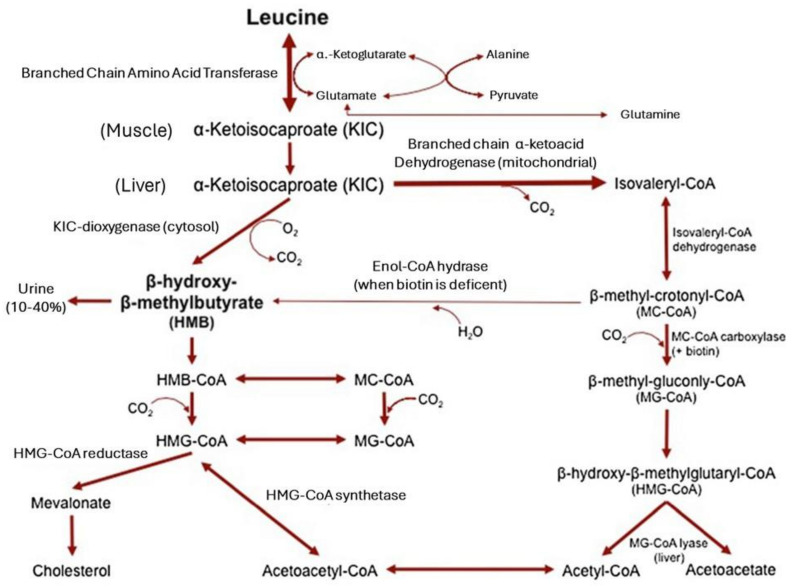
The metabolism of beta-hydroxy-beta-methyl-butyrate. Reproduced with permission from Wilson et al. [149].

β-hydroxy-β-methylbutyrate (HMB) is a molecule derived from the metabolism of leucine (Figure 4), which is able to stimulate muscle protein synthesis and suppress muscle protein breakdown [144]. However, only about 5% of leucine is converted into HMB, which makes the natural levels of HMB quite low after the ingestion of leucine [145]. The recommended dose of HMB to stimulate muscle anabolism is 3 g per day, so that 60 g of leucine are necessary to obtain the required amount of HMB [146]. Since this is practically impossible through the diet, a proper HMB supplementation is necessary to achieve anabolic and anti-catabolic effects, especially in the context of sarcopenia or muscle recovery. HMB supplementation has also been tested in older adults in whom growing clinical evidence suggests that it can slow down muscle loss and improve muscle strength [147,148]. The mechanisms through which HMB works are multiple, and they involve both anabolic and anti-catabolic processes.

HMB primarily works by inhibiting protein degradation pathways, particularly the ubiquitin–proteasome system, which plays a key role in muscle loss. This is especially beneficial during conditions of physical stress, such as prolonged immobilization, periods of inactivity, chronic diseases, or in older adults suffering from sarcopenia [150].

As previously mentioned, HMB activates protein synthesis via the mTOR (mechanistic target of rapamycin) pathway, which represents the main central anabolic signaling pathway for muscle growth. Stimulation of mTORC1 through a leucine-independent sensing pathway promotes the accumulation of new proteins within muscle tissue, counteracting muscle mass loss and aiding in muscle repair and regeneration [144,151]. This anabolic action helps maintain or improve muscle function, especially during periods when muscle repair is crucial.

Another mechanism mediating the beneficial effects of HMB on muscle protein synthesis is its anti-inflammatory effects and reduction in oxidative stress, both of which contribute to muscle degradation. Chronic inflammation and oxidative damage are major factors that accelerate muscle mass loss in aging individuals and patients with chronic conditions. By reducing the levels of pro-inflammatory cytokines, like TNF-α and IL-6, HMB can help protect against muscle degradation caused by chronic inflammation. Additionally, HMB reduces the activation of pro-inflammatory pathways, such as NF-κB, which plays a significant role in promoting muscle catabolism by inducing the breakdown of muscle proteins [152].

Finally, HMB may be an effective supplement for maintaining muscle mass, strength, and function, particularly in the elderly and in patients experiencing conditions that predispose them to muscle loss, such as chronic diseases, physical inactivity, or aging. The modulation of mitochondrial activity and lipid metabolism may be involved in preventing disuse atrophy and rehabilitation, two mechanisms that go beyond HMB’s effects on muscle protein synthesis and degradation [151].

#### 8.1.3. Omega-3 Fatty Acids

Clinical studies on the effectiveness of *n*-3 PUFA supplementation in increasing muscle mass have yielded conflicting results. This may be due to differences in study population, design, and outcome measurements. Nevertheless, a recent meta-analysis that included eight RCTs investigating the effect of *n*-3 PUFA on muscle synthesis concluded that, even if *n*-3 PUFA supplementation has no effect on muscle protein synthesis, they promoted whole-body protein synthesis in healthy and ill adults [153]. It should be considered that most of the participants were healthy subjects, and only two studies included patients with chronic diseases; moreover, the quantity of *n*-3 PUFA and the ratio between eicosapentaenoic acid (EPA) and docosahexaenoic acid (DHA) varied in the different studies (2.97–0.6 g EPA and 2.28–0.6 g DHA and 3.5–0.68 EPA/DHA, respectively). Notably, *n*-3 PUFA supplementation appears to be more beneficial for individuals with chronic inflammation and cachexia rather than healthy adults, regardless of the participants’ age, dosage, duration of supplementation, or engagement in resistance exercise. Interestingly, *n*-3 PUFA supplementation would not directly increase muscle protein synthesis but could act by augmenting the mTORC-1 signaling responses to other stimuli, such as hyperaminoacidemia and hyperinsulinemia [154]. Additional studies on the mechanism of action related to omega-3 fatty acids suggest an enhanced feeding-induced protein synthesis and a downregulation of myostatin and Foxo1 [155,156]. Finally, data support the beneficial effects of omega-3 fatty acids on muscle metabolism, primarily through their anti-inflammatory activity [155,157,158,159]. Engelen et al. [155] suggested to administer the capsules containing omega-3 fatty acids at lunch and breakfast to maximize their anabolic response.

#### 8.1.4. Vitamin D

The expression of vitamin D receptors (VDRs) declines with aging [160]. As a result, other aspects of vitamin D metabolism beyond its influence on calcium homeostasis have received attention. Thanks to the activation of its receptor expressed by muscle cells, vitamin D regulates the transcription of genes involved in muscle cell differentiation and proliferation [161]. Moreover, there is a positive correlation between serum concentration of 25(OH)D and muscle function.

In a study by Okuno et al. [162] conducted on 80 elderly Japanese women aged > 65 years, vitamin D insufficiency or deficiency was observed in 89% and 28% of the cases, respectively. Among this group, 56.3% experienced falls during a three-month observation period. Moreover, a meta-analysis including 10 randomized controlled trials involving 2426 subjects (mean age 80 years, 81% women) on the effects of vitamin D supplementation on falls and bone fractures reported that vitamin D supplementation reduced the risk of falls by 19–22% in those subjects assuming a dose of 700–1000 IU daily [163]. Finally, a longitudinal observational study in subjects aged > 65 years concluded that elderly individuals with low serum levels of vitamin D and high levels of parathyroid hormone are more susceptible to sarcopenia [164].

Severe vitamin D deficiency is known to be associated with muscle pain and significant weakness. In adults experiencing severe deficiency, muscle biopsies reveal atrophy of type II muscle fibers, resembling the changes observed in aging. Vitamin D receptors (VDRs), found in muscle tissue, tend to decrease with age [165,166]. Several intervention trials have been conducted to investigate the effects of vitamin D supplementation on muscle function, leading to conflicting results. A meta-analysis by Stockton et al. [167] examined 17 randomized clinical trials (RCTs) involving 5072 adults and assessed the impact of vitamin D supplementation on muscle performance. They concluded that there was no benefit in terms of muscle strength for participants with a baseline 25(OH)D serum level above 25 nM. However, in the two studies that included participants with 25(OH)D levels < 25 nM, a significant increase in muscle strength was observed. More recent data suggest that 25(OH)D supplementation may benefit muscles in elderly individuals with levels < 40 nmol/L [165]. The benefits of vitamin D supplementation are not limited to older or frail populations. In this regard, a meta-analysis of seven studies, which included six RCTs and one controlled trial, including 310 participants aged 21.5–31.5 years, found that high doses of vitamin D (ranging from 4000 IU per day to 60,000 IU per week for 1–6 months) resulted in an improvement in both upper and lower limb muscle strength [168]. However, this effect was not observed in another meta-analysis of 16 randomized controlled trials investigating the effects of vitamin D supplementation on muscle function in 7765 post-menopausal women [169]. In this case, the duration of the treatment varied from 4 to 96 weeks, and different dosages of vitamin D were used alone or associated with calcium and alendronate. Therefore, the different effects reported on vitamin D supplementation may be due to various factors such as the amount and type of vitamin D used and the duration of the intervention and the vitamin D status of the participants [170]. Consequently, there is still no standardized cutoff for vitamin D levels to definitively establish sufficiency [171].

Although few foods naturally contain vitamin D, fatty fish (trout, salmon, tuna, and mackerel) and fish liver oils are among the best sources [172,173]. Beef liver, egg yolks, and cheese contain small amounts of vitamin D, primarily in the form of vitamin D3 and its metabolite 25(OH)D3. Interestingly, fortified foods now provide most of the vitamin D in American diets [173,174].

### 8.2. Foods for Special Medical Purposes

Food for special medical purposes (FSMPs) are nutritional products specifically formulated to manage the specific nutritional needs of individuals with certain diseases or clinical conditions. These products are intended to supplement or replace the normal diet in patients who, for clinical reasons, cannot obtain the necessary nutrients from standard food sources [175]. FSMPs are classified into three groups: (a) nutritionally complete foods with a standard nutritional formulation, (b) nutritionally complete foods with a disease-specific formulation, and (c) foods lacking one or more components, with a formulation adapted for a disease or medical condition. The first two categories may serve as the sole source of nutrition for a patient or be used to reach the daily intake requirements, while the third can only supplement other nutrient sources [176].

In the context of sarcopenia, FSMPs aim to support the preservation and increase in muscle mass and strength, especially in elderly individuals and those with chronic diseases, particularly if associated with chronic inflammation and corticosteroid use [177]. An interesting unpublished study conducted by our group evaluated the effect of a supplement rich in branched-chain amino acids (BCAAs) and HMB administered for three months to patients with rheumatological disease and sarcopenia. Preliminary results indicated statistically significant improvements in terms of strength and muscle mass, suggesting the potential benefit of such nutritional formulations in counteracting the muscle decline associated with chronic inflammatory conditions.

FSMPs for treating sarcopenia mainly consist of branched-chain amino acids, often combined with HMB and vitamin D. These components play a crucial role in reducing catabolism, improving insulin sensitivity, and stimulating protein synthesis [178]. In addition to protein components, some FSMPs include antioxidants and micronutrients like zinc and magnesium, which reduce oxidative damage and inflammation, thereby improving the metabolic environment of skeletal muscle [179]. Another frequently used component is represented by omega-3 polyunsaturated fatty acids that show anabolic effects on muscle metabolism, further supporting protein synthesis and muscle function [180].

There is significant scientific evidence supporting these nutritional interventions, as highlighted in various meta-analyses. Nasimi et al. [181] evaluated thirty RCTs on the effectiveness of whey protein supplementation either alone (22 studies) or in association with vitamin D (eight studies) on lean mass, muscle strength, and physical function in subjects aged > 60 years, with or without sarcopenia or frailty, and in the absence of diseases potentially influencing the outcome. Different dosages of whey proteins (15–40 g/day) and vitamin D (100–800 IU) were used for more or less than 12 weeks. Whey proteins, especially at the dosage > 20 g/day, significantly improved appendicular lean mass in elderly individuals with sarcopenia or frailty but were not effective in healthy subjects. On the other hand, the combination of whey protein and vitamin D produced more pronounced benefits, even in the short-term studies (less than 12 weeks), not only in frail or sarcopenic individuals but also in healthy subjects, probably through a correction of low vitamin D levels. However, due to the high heterogeneity of the studies, the overall quality of the evidence was mostly low or very low. Another meta-analysis conducted by Cuyul-Vásquez et al. [182] analyzed seven studies investigating the effectiveness of whey protein supplementation in 591 elderly individuals with sarcopenia undergoing a resistance exercise program aimed at improving skeletal muscle mass and strength. The authors concluded that, although whey protein supplementation during resistance exercises had positive effects on muscle mass and strength, the effects were modest, and the quality of the evidence was low.

A systematic review of scientific literature by Cereda et al. [183] analyzed 10 studies on the beneficial effects of specific oral nutritional supplementation (muscle-targeted oral nutritional supplementation—MT-ONS) based on whey protein, leucine, and vitamin D. Their findings support the use of MT-ONS as a first-line therapeutic option or in combination with physical exercise as a strategy for ameliorating muscle mass, strength, and physical performance in the elderly with sarcopenia or at risk of muscle loss [183]. However, two of the studies did not evaluate the impact of MT-ONS on muscle mass and strength; instead, they focused on the effect on inflammation or the relationship between vitamin D levels and muscle mass. Moreover, three studies conducted in different community settings found no effect on muscle strength. Among the last four studies, which took place in long-term care setting and rehabilitation centers, only two reported an improvement in muscle strength. Furthermore, the results did not determine the optimal duration of the intervention, its effectiveness in other clinical settings, or the total energy intake required.

A common element emerging from the various meta-analyses is the effectiveness of the integrated approach combining whey protein, vitamin D, and resistance training. A crucial aspect highlighted in the various meta-analyses is the importance of the optimal dosage of whey proteins. Doses above 20 g appear to be more effective in enhancing the effects of resistance training, suggesting that, to achieve significant improvements in muscle strength and lean mass, the amount of protein consumed must be carefully monitored.

## 9. Disease Specific Aspects and Nutritional Treatment

### 9.1. Nutritional Treatment in Cancer Patients

It is well known that maintaining good nutritional status improves prognosis, including tolerance to pharmacological treatment and survival, in cancer patients [184]. In these patients, it is crucial to assess energy requirements since optimal energy intake levels are needed not only to avoid weight loss but also to preserve muscle mass by stimulating protein synthesis and suppressing protein degradation [184]. Resting energy expenditure (REE) in cancer patients is determined by the type of cancer [185], and it has been shown that REE is significantly elevated in patients with an acute-phase response [61]. However, although REE may be elevated in cancer patients, total energy expenditure (TEE) is often decreased due to a reduced physical activity level (PAL) [185]. In a study of patients with hypermetabolic and cachectic pancreatic cancer, it was shown that the measured average PAL was much lower (mean 1.24) than that recorded in healthy adults of similar age (mean 1.62) [186]. Consistent with these data, a study conducted on patients with gastrointestinal tumors undergoing chemotherapy and following accurate nutritional monitoring showed that the TEE measured with an advanced fitness tracker remained stable throughout treatment in patients with a Karnofsky PS > 50. This suggests that chemotherapy does not significantly alter either REE or physical activity in the absence of significant weight loss [187].

The current energy recommendations by ESPEN are based solely on body weight and suggest a caloric intake of 25–30 kcal/kg/day [188]. However, it is important to note that the protein intake guidelines for cancer patients are not specifically targeted at managing reduced muscle mass but provide a general recommendation ranging from 1.0 to 1.5 g of protein per kilogram of body weight per day [189], emphasizing that these guidelines do not necessarily consider the specific needs of individuals with muscle loss but offer a general range to meet protein requirements. Furthermore, guidelines on protein intake based only on body weight do not account for the significant variability in body composition among populations [190].

Among patients with active diseases, a review of studies on cancer cachexia concluded that dietary proteins > 1.5 g/kg/day can maintain or improve muscle mass, and these effects may be more substantial when combined with exercise in other clinical populations with cachexia [191]. However, the quality of protein sources plays an essential role, as suggested by Ford KL et al. [192], who emphasized the importance of including animal-based proteins, such as fish, eggs, milk, and cheese, to support muscle anabolism in cancer patients. This work recommended that 65% of the daily protein intake should come from animal sources as a starting point, with careful planning of substitutions for animal products to avoid nutritional deficiencies.

Another aspect to consider is the timing of protein distribution (109). A literature review [193] suggests that the quantity of amino acids necessary to maintain a positive protein balance in cancer patients may be close to 2 g/kg/day. This is supported by a study conducted by Winter et al. [142], which examined moderately cachectic lung cancer patients who exhibited significant insulin resistance, being that normal insulin sensitivity is required for optimal muscle protein anabolism [142,194]. The condition of hyperaminoacidemia was able to restore a normal anabolic response of body proteins, suggesting the therapeutic potential of a high amino acid intake. This strategy applies to individuals with normal kidney functions who can safely assume a protein intake of up to 2 g/kg/day or more [195].

An additional strategy in the diet of cancer patients emphasizes the integration of eicosapentaenoic acid (EPA), which could help improve appetite, food intake, body weight, and muscle mass in individuals at risk of body composition alterations [196]. In this context, nutritional counseling is a key tool to ensure that cancer patients with a functioning gastrointestinal tract receive an adequate and personalized diet [197]. What emerges from the literature is that nutritional practice should aim to provide all patients with a nutritionally adequate diet to combat malnutrition, which also includes all micronutrient classes [198].

The recommendations from the American Cancer Society also suggest the use of multivitamin–multimineral supplements in physiological doses, meaning the amounts of the nutrients approximately correspond to the recommended daily allowances [199]. Therefore, the role of the dietitian–nutritionist is crucial in providing individualized advice to balance the patient’s energy and nutritional intake, considering their specific needs; the presence and severity of symptoms, such as anorexia, nausea, dysphagia, bloating, abdominal cramps, diarrhea or constipation; and any integration requirements [196].

Nutrition is essential in cancer care and should be considered from the moment of diagnosis and integrated into the therapeutic pathway [188]. It should include specific recommendations based on the type of cancer, such as increasing the proportion of energy derived from fats compared to carbohydrates in patients with insulin resistance and reducing the risks related to hyperglycemia in cases of parenteral nutrition. Artificial nutrition or nutrition with ONS is also an integrative approach to combat malnutrition and weight loss [200,201,202], aiming to adopt the least invasive strategy possible while balancing the nutritional benefits with the risks and impact on quality of life [203].

### 9.2. Nutrition in Kidney Diseases and Cirrhosis

Chronic kidney failure and liver cirrhosis require specific nutritional strategies to preserve muscle function and improve the quality of life of patients. In patients with acute or chronic kidney failure, protein intake should not exceed 1.0 or 1.2 g/kg/day, respectively [204]. Effective nutritional management for chronic kidney failure must be personalized, considering the patients’ specific needs according to the disease stage, treatment modality, and any existing comorbidities [205]. For nondiabetic patients with chronic kidney disease stages III–IV, a protein intake of 0.55–0.60 g/kg/day is recommended. It is crucial that most of the proteins come from high-value sources, such as eggs, dairy, meat, and fish, to ensure adequate intake of essential amino acids. When protein intake is significantly reduced, the current guidelines recommend using α-keto analogs of amino acids to prevent nutritional deficiencies and reduce nitrogen waste production and urinary pH. For patients undergoing dialysis, who lose protein during treatment, a protein intake of 1.0–1.2 g/kg/day is recommended, while a protein intake of 1.2–1.3 g/kg/day is recommended for those on peritoneal dialysis. In the immediate period after kidney transplant, a higher protein intake is necessary to support healing and anabolism: 1.2–1.5 g/kg/day. Once stability is achieved, protein intake can be adjusted to levels similar to those recommended for the general population, around 0.8–1.0 g/kg/day [206]. Inadequate protein intake, especially in patients with advanced chronic kidney disease, can promote muscle mass loss and sarcopenia, negatively affecting quality of life and clinical outcomes. Therefore, careful nutritional management is essential to mitigate the catabolic effects of kidney disease and preserve muscle function.

Similarly to what is observed in cancer patients, sarcopenia due to liver cirrhosis is one of the most significant complications, with a negative impact on prognosis and quality of life. Muscle mass loss in these patients is caused by various factors, including metabolic alterations, chronic inflammation, and malnutrition. Liver cirrhosis is considered an “accelerated starvation state” in which the body rapidly enters a post-absorptive phase, relying on its energy reserves to sustain metabolism and mobilize fat stores [207]. This state is characterized by a reduced “respiratory quotient” [208]. Furthermore, during this accelerated starvation state, protein synthesis decreases, and gluconeogenesis from amino acids increases, leading to proteolysis and sarcopenia. Most nutritional intervention studies in liver cirrhosis recommend a caloric intake of at least 35 kcal/kg/day and a protein intake of 1.2–1.5 g/kg/day to prevent or counterbalance muscle mass loss [207]. Additionally, because cirrhosis is characterized by this “accelerated starvation state”, evidenced by rapid activation of post-absorptive mechanisms and a reduction in the respiratory quotient, recent studies have assessed the effectiveness of frequent feeding to prevent the effects of prolonged fasting, particularly during the night. Since overnight fasting represents the longest interval between meals, strategies such as introducing a late evening snack have shown benefits in improving the metabolic profile and enhancing the quality of life for patients, although the impact on muscle mass has not always been consistent [209]. It is recommended that cirrhotic patients adopt a diet regimen that includes six meals per day, including a protein-rich breakfast and an evening snack, to reduce the fasting period and limit negative metabolic effects [210,211]. However, achieving adequate caloric and protein intake can be challenging in malnourished sarcopenic patients with advanced liver disease, especially if there is decompensation [212]. In these situations, oral BCAA supplements have been used in clinical studies to address this challenge [213,214], or 24 h continuous low-volume/low-calorie enteral nutrition via nasogastric tube has also shown some promising benefits, even during repeated episodes of decompensation [211].

## 10. Future Perspectives

Currently, strength exercise training and nutritional treatments are the only widely accepted treatments for sarcopenia, although numerous alternative strategies have been proposed in the past. These treatments included hormones (testosterone, estrogens, IGF-1, GH, irisin, etc.), the glycoprotein follistatin and the proinsulin-C-peptide, as well as several drugs (myostatin blockers, ACE, ARB, beta-blockers) [108,215]. However, it should be underlined that strength exercise training and nutritional treatments are efficacious against sarcopenia induced by malnutrition or physical inactivity but only partially counteract the sarcopenia due to inflammation and metabolism dysregulation that occurs in chronic inflammatory diseases and cachectic patients. As previously illustrated, a potential new approach to the treatment of sarcopenia could be represented by probiotics, which have given promising results in patients with obesity or metabolic syndrome. Moreover, innovative research methodologies, such as the application of artificial intelligence (AI) and pharmacological intervention with molecular targeting, could generate further impulse to research in this field.

## 11. Limitations and Conclusions

As for narrative reviews in general, in our search, we did not include all relevant literature on the considered topics. However, starting from the selected articles, we found other articles that allowed for us to study the various topics in depth. In addition, we should consider that narrative reviews do not include all literature addressing the phenomenon of interest.

Experimental studies conducted by Kucharski et al. [216] have shown that diet can lead to significant epigenetic changes, which can even influence development in adulthood. This indicates that nutrition is vital in the treatment of sarcopenia and the prevention of age-related decline. The nutritional treatment of secondary sarcopenia, especially in cancer patients, appears to be a more complex challenge. Targeted nutrition, which includes amino acids, high-quality proteins, essential fatty acids, and the appropriate intake of vitamins and micronutrients, is crucial for supporting muscle protein synthesis, reducing muscle mass loss, and promoting overall well-being. However, it remains to consider the necessity to individualize nutritional therapy based on the underlying disease.

## Figures and Tables

**Table 1:** Criteria for the diagnosis of sarcopenia.

Diagnostic Phase	Main Parameter	Description	Type of Measurement
Suspected sarcopenia	Muscle strength	The reduction is an early indicator of sarcopenia.	Handgrip test
Confirmation of sarcopenia	Muscle mass	The reduction confirms the diagnosis of sarcopenia.	DEXA, BIA
High-grade sarcopenia	Physical performance	When it is associated with a reduction in function and mobility.	Walk speed test

**Table 2:** Classification of sarcopenia. Modified from Cruz-Jentoft et al. [5].

Type of Sarcopenia	Methods of Identification
Primary Sarcopenia	
Age-related sarcopenia	Absence of other causes.
Secondary Sarcopenia	
Sarcopenia related to physical activity	Sedentary lifestyle, bed rest syndrome.
Sarcopenia related to diseases	From advanced organ failure, inflammatory disease, neoplasia, or endocrine disease.
Sarcopenia related to nutritional aspects	From inadequate energy and/or protein intake, intestinal malabsorption, or anorexia.

## Supplementary Materials

The following supporting information can be downloaded at: https://www.mdpi.com/article/10.3390/nu17071237/s1, Table S1: Search strategy for PubMed and Scopus; Table S2: Search strategy for WOS.

## Abbreviations

The following abbreviations are used in this manuscript:

PI3K	phosphoinositide-3-kinase
IGF-1	insulin-like growth factor
PIP3	phosphoinositide-3,4,5-trisphosphate
PDK1	phosphoinositide-dependent kinase 1
FoxO	Forkhead Box
mTOR	mammalian target of rapamycin
MuRF1	muscle ring finger 1
MAFbx	muscle atrophy F-box
mTORC1	mTOR Complex 1
mTORC2	mTOR Complex 2
GDF-8	growth and differentiation factor 8
TGF-β	transforming growth factor beta
TNF-α	tumor necrosis factor alpha
CIFs	cachexia-inducing factors
UCP1	uncoupling protein 1
LMF	lipid-mobilizing factors
HMB	β-hydroxy-β-methylbutyrate
FMSPs	food for special medical purposes
MT-ONS	targeted oral nutritional supplementation
PAL	physical activity level
REE	resting energy expenditure
TEE	total energy expenditure
EPA	eicosapentaenoic acid
