# Supplementary material for: An Overview of Sarcopenia: Focusing on Nutritional Treatment Approaches
Source: Nutrients. 2025 Apr 1;17(7):1237. doi: 10.3390/nu17071237 (PMC11990658; doi:10.3390/nu17071237)
Supplement: Supplementary file 1 [file nutrients-17-01237-s001.zip › Supplementary Tables S1 and S2.pdf]

Supplementary Materials

Table S1. Search strategy for PubMed and Scopus.

(sarcopenia[Title]) AND (definition[Title]), with "Review" applied as filter;
sarcopenia[Title]) AND (definition[Title]) AND classification;
(sarcopenia[Title]) AND (epidemiology[Title]);
(sarcopenia[Title/Abstract]) AND (pathophysiology[Title/Abstract]) AND (aging[Title/Abstract]);
(sarcopenia[Title/Abstract]) AND ("sex hormone*"[Title/Abstract]) with "Review" applied as filter;
("cancer-related sarcopenia"[Title/Abstract]);
(sarcopenia[Title/Abstract]) AND ("Branched-chain amino acid*"[Title/Abstract]) with "Review" applied as filter;
(sarcopenia[Title/Abstract]) AND ("β-Hydroxy-β-methylbutyrate"[Title/Abstract]) with "Review" applied as filter;
(sarcopenia[Title/Abstract]) AND ("whey protein*"[Title/Abstract]) with "Review" applied as filter;
(sarcopenia[Title/Abstract]) AND ("Omega-3 fatty acid*"[Title/Abstract]) with "Review" applied as filter;
(sarcopenia[Title/Abstract]) AND ("vitamin D"[Title/Abstract]) with "Systematic Review" applied as filter;
(sarcopenia[Title/Abstract]) AND ("Food for Special Medical Purposes"[Title/Abstract]) OR FSMPs;
(sarcopenia[Title/Abstract]) AND ("liver cirrhosis"[Title/Abstract]) with "Systematic Review" applied as filter;
(sarcopenia[Title/Abstract]) AND ("renal disease*"[Title/Abstract]) with "Review" applied as filter;

Table S2. Search strategy for WOS.

(sarcopenia) AND (definition) filtered by "Title, and refined by "Review"
(sarcopenia) AND (definition) AND classification
(sarcopenia) AND (epidemiology) filtered by "Title
(sarcopenia) AND (pathophysiology) AND (aging) filtered by "Title or Abstract" and refined by "Review"
(sarcopenia) AND ("sex hormone*") filtered by "Title or Abstract"
("cancer-related sarcopenia") filtered by "Title or Abstract"
(sarcopenia) AND ("Branched-chain amino acid*") filtered by "Title or Abstract" and refined by "Review"
(sarcopenia) AND ("β-Hydroxy-β-methylbutyrate") filtered by "Title or Abstract" and refined by "Review"
(sarcopenia) AND ("whey protein*") filtered by "Title or Abstract" and refined by "Review"
(sarcopenia) AND ("Omega-3 fatty acid*") filtered by "Title or Abstract" and refined by "Review"
(sarcopenia) AND ("vitamin D") filtered by "Title or Abstract" and refined by "Systematic Review"
(sarcopenia) AND ("Food for Special Medical Purposes"[Title/Abstract]) OR FSMPs;
(sarcopenia[Title/Abstract]) AND ("liver cirrhosis"[Title/Abstract]) with "Systematic Review" applied as filter;
(sarcopenia[Title/Abstract]) AND ("renal disease*"[Title/Abstract]) with "Review" applied as filter;
